# Testing alternative hypotheses on the origin and speciation of Hawaiian katydids

**DOI:** 10.1186/s12862-022-02037-2

**Published:** 2022-06-22

**Authors:** Mohan Rakesh, Stephane Aris-Brosou, X. Xia

**Affiliations:** 1 Department of Biology, University of Ottawa, 30 Marie Curie, Station A, P.O. Box 450, Ottawa, ON K1N 6N5 Canada; 2 Department of Mathematics and Statistics, University of Ottawa, Ottawa, ON K1N 6N5 Canada; 3 Ottawa Institute of Systems Biology, Ottawa, ON K1H 8M5 Canada

**Keywords:** Phylogeography, Biogeography, Geophylogeny, Speciation, DNA barcoding, Phylogenetics, Hawaii Islands

## Abstract

**Background:**

Hawaiian Islands offer a unique and dynamic evolutionary theatre for studying origin and speciation as the islands themselves sequentially formed by erupting undersea volcanos, which would subsequently become dormant and extinct. Such dynamics have not been used to resolve the controversy surrounding the origin and speciation of Hawaiian katydids in the genus *Banza*, whose ancestor could be from either the Old-World genera *Ruspolia* and *Euconocephalus*, or the New World *Neoconocephalus.* To address this question, we performed a chronophylogeographic analysis of *Banza* species together with close relatives from the Old and New Worlds.

**Results:**

Based on extensive dated phylogeographic analyses of two mitochondrial genes (*COX1* and *CYTB*), we show that our data are consistent with the interpretation that extant *Banza* species resulted from two colonization events, both by katydids from the Old World rather than from the New World. The first event was by an ancestral lineage of *Euconocephalus* about 6 million years ago (mya) after the formation of Nihoa about 7.3 mya, giving rise to *B. nihoa.* The second colonization event was by a sister lineage of *Ruspolia dubia.* The dating result suggests that this ancestral lineage first colonized an older island in the Hawaiian–Emperor seamount chain before the emergence of Hawaii Islands, but colonized Kauai after its emergence in 5.8 mya. This second colonization gave rise to the rest of the *Banza* species in two major lineages, one on the older northwestern islands, and the other on the newer southwestern islands.

**Conclusion:**

Chronophylogeographic analyses with well-sampled taxa proved crucial for resolving phylogeographic controversies on the origin and evolution of species colonizing a new environment.

**Supplementary Information:**

The online version contains supplementary material available at 10.1186/s12862-022-02037-2.

## Background

Phylogeographic studies of biodiversity in remote islands, such as Hawaii Islands, offer several unique opportunities for studying the origin and evolution of invasive species in a new environment. First, the islands have a well-known origin through volcanic eruptions with a well dated history [1–3] providing an upper bound of time for colonization events. Second, the geographic location of the islands and their distance of approximately 3850 km from the mainland or French Polynesia [4] implies a rarity of successful colonization events, which probably occurred during glacial periods of the earth when much of ocean water was locked on land in the form of thick icesheets so that many submarine islands were above sea water to serve as bridges of insect migration. For example, the ice sheet in Antarctica expanded to its maximum around 5.6 mya, contributing to the decrease in sea level and the complete drying-up of the Mediterranean Sea [5]. The rarity of long-distance migration over the ocean implies that the descendent lineages on Hawaii Islands are unlikely to disperse back to their ancestral or other mainland regions, especially if they become flightless soon after their arrival on the islands (discussed later). This conceptually simplifies the identification of the ancestral lineage. Third, the difference in habitats between the ancestors (typically on the mainland) and the descendants on the islands imposes differential selection and adaptation, leading to rapid phenotypic diversification on the island lineages in a short time [6], presumably aided by the small founding population that allowed the island lineage to escape a local fitness peak constrained by epistatic interactions. Fourth, geographic isolation among the islands and the associated rarity in inter-island gene flow lead to genetic isolation and speciation events that are relatively easy to detect and characterize by molecular phylogenetics and phylogeography [7, 8].

To demonstrate the power of such approaches, we here focus on the origin and speciation of the ten endemic Hawaiian katydids in the Genus *Banza*. This group of insects has become flightless with reduced wings. This limitation on their dispersal ability enhanced the genetic isolation and speciation process on the individual islands. In contrast, a winged grasshopper such as *Schistocerca nitens* was capable of dispersing from Hawaii to Nihoa by trade winds [9]. Both trade winds and extreme storms appear to have contributed to the observed biogeographic patterns in Hawaii Islands [10].

The pioneering study of Hawaiian katydids by Shapiro et al. [11] aimed to address four key questions associated with the origin and speciation of these flightless katydids. How many colonization events had occurred that resulted in subsequent origin and speciation of the katydids? Do the *Banza* species form a monophyletic taxon associated with a single colonization event? What is the ancestral lineage (or lineages) from which the Hawaiian katydids were derived? Are the ancestral lineages from the coastal region of eastern Asia or western coast of America? These questions are closely related and answering one of them will shed light on the others. However, the limitation of sampling in that study, as discussed by Shapiro et al. [11], did not allow these questions to be fulling answered.

Three katydid genera, all featuring strong fliers, have been suggested Shapiro et al. [11] as potential ancestral lineages of the *Banza* species: *Euconocephalus* and *Ruspolia* in the Old World, and *Neoconocephalus* in the New World. Therefore, determining the ancestral lineage also provides information on the ancestral region of the *Banza* species. The study by Shapiro et al. [11] includes *Neoconocephalus triops* and two other North American species that diverged before the common ancestor of *Neoconocephalus* and *Banza* species, but no species from the Old World such as *Euconocephalus* or *Ruspolia* which is acoustically similar to *Neoconocephalus* and *Banza* species [12]. These two Old World genera were also missing in a recent phylogenetic study involving *Neoconocephalus* species [13]. Consequently, such studies cannot discriminate between the two alternative origin hypotheses, i.e., from *Euconocephalus* or *Ruspolia* lineages in the Old World or from *Neoconocephalus* in the New World.

**Fig. 1:**
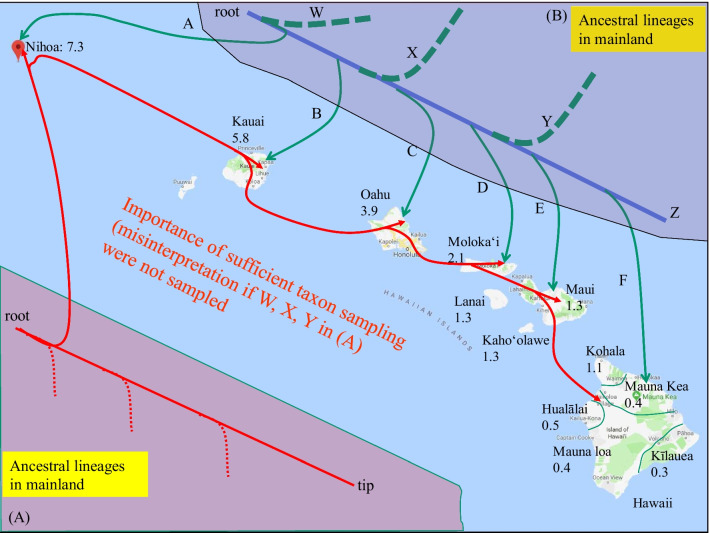
Hawaiian Islands with inferred approximate time on their volcanic origin in million years [2, 14], together with a depiction of **A** repeated successful colonization of the islands by the ancestral lineages, and **B** a single successful colonization followed by island hopping by the founding lineage in Nihoa. This figure was hand-drawn on a google map

Complete mitochondrial genomes are now available for *Euconocephalus nasutus* (the type species) and *E. pallidus,* as well as for *Ruspolia dubia* and *R. lineosa.* Their inclusion in the phylogeographic analysis is essential because insufficient taxon sampling may lead to misleading conclusions, as discussed in Shapiro et al. [11]. We contrast two extreme hypotheses in Fig. 1. The red lines depict a single colonization followed by successive island-hopping events that occurred as new islands emerged (Fig. 1A). This scenario is consistent with the phylogenetic results in Shapiro et al. [11], with the *Banza* species forming a monophyletic clade. The green lines represent a scenario with repeated colonization events from the mainland, but each colonization event was followed by wing loss before the emergence of the next island, so the flightless katydids could not colonize new islands that emerge through later volcano activities (Fig. 1B). If mainland lineages X, Y and Z in Fig. 1B were never sampled, then all island species would superficially constitute a monophyletic taxon, with lineage W misidentified as the closest phylogenetic relative of all island species. Consequently, the scenario in Fig. 1B would be indistinguishable from that in Fig. 1A. For this reason, Shapiro et al. [11] cautioned a simple-minded interpretation of their phylogenetic tree which superficially suggests that Hawaiian katydids were derived from a single colonization event by an ancestor closely related to *Neoconocephalus triops*.

We performed phylogenetic analysis of the *Banza* species with the addition of species from *Euconocephalus* and *Ruspolia*, with extensive likelihood-based and Bayesian inference to improve on the previous phylogenetic analysis [11] without *Euconocephalus* and *Ruspolia* species. Our results suggest two successful colonization events that resulted in the extant *Banza* species. The first colonization event occurred about 6 mya by an ancestor of *Euconocephalus,* which gave rise to *B. nihoa.* The second colonization event occurred indirectly. An ancestor closely related to *Ruspolia dubia* colonized an island in the Hawaiian–Emperor seamount chain around 8.8 mya before the emergence of Hawaii Islands, and subsequently colonized Kauai and the younger islands after their sequential emergence. This gave rise to all other *Banza* species. These *Euconocephalus* and *Ruspolia* lineages inhabit eastern and southeastern Asia, including the coastal regions, so the ancestral lineage of *Banza* species is inferred to be from Asia instead of from America.

## Results

### Hawaiian katydids descended from Old World copiphorines in two colonization events

**Fig. 2:**
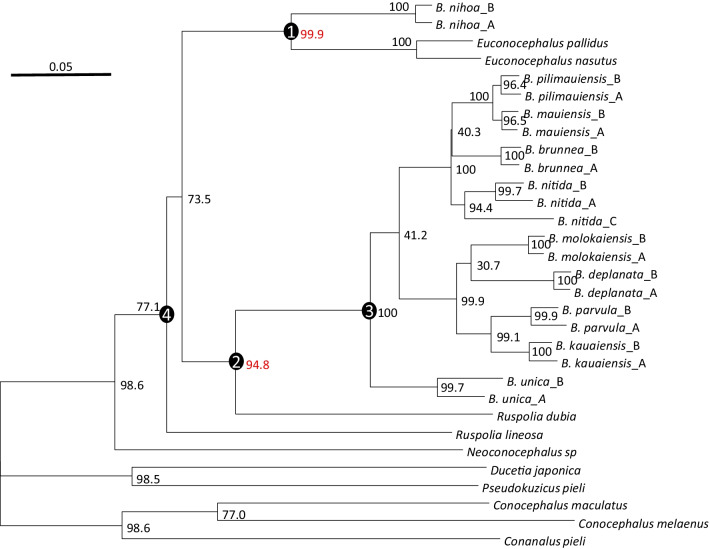
Phylogenetic trees from PhyML analysis. Bootstrap support values are shown near internal nodes, with the two key support values (99.9 and 94.8) associated with the two colonization events highlighted in red. The RAxML tree has identical topology and nearly identical branch lengths, but with slightly lower bootstrap values. Four key internal nodes are numbered and referred to in the text as Nodes 1, 2, 3 and 4, respectively

**Fig. 3:**
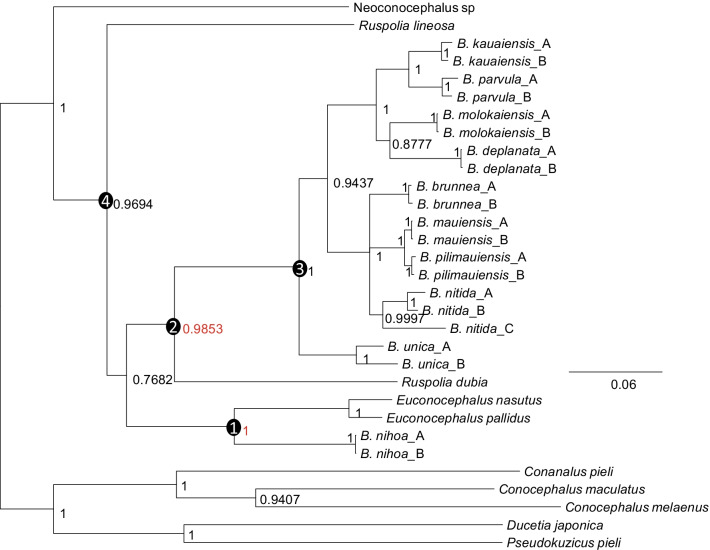
Phylogenetic trees from Bayesian analysis with MrBayes, with GTR + Γ + I as substitution model. Posterior probabilities are next to internal nodes, with the two key posterior probabilities (0.9853 and 1) supporting the two colonization events highlighted in red. The two numbers are 0.9880 and 1, respectively, when running MrBayes with GTR + Γ as substitution model. The four numbered internal nodes are equivalent to those in Fig. 2

Our phylogenetic reconstructions with maximum likelihood (PhyML and RAxML) produced the same topology and nearly identical branch lengths (Fig. 2), which is expected because the same substitution model (GTR + Γ) has been used for both PhyML and RAxML. The PhyML tree is shown (Fig. 2). The topology from the two likelihood-based methods is also identical to that of the Bayesian tree from MrBayes (Fig. 3). The branch lengths of the Bayesian tree differ from that of those of the likelihood tree because of a slightly different substitution model (GTR + Γ + I) was used for MrBayes. The subtree support indicated by the posterior probabilities from the Bayesian inference (Fig. 3) appears higher than by the bootstrap support from the likelihood method (Fig. 2).

The trees in Figs. 2, 3 show that the ancestors of Hawaiian katydids are copiphorines in the Old World, related to *Ruspolia* and *Euconocephalus*. A previous study including only *Neoconocephalus triops,* but not *Ruspolia* and *Euconocephalus*, suggests that the ancestor of Hawaiian katydids is closely related to New World copiphorines [11, 15]. However, the original authors emphasized that the ancestor of Hawaiian katydids are equally likely to be from *Ruspolia* and *Euconocephalus* [11, 15]. Our results have now resolved this uncertainty: not only are Hawaiian katydids derived from Old World copiphorines, but there are also two independent colonization events leading to extant Hawaiian katydid lineages, both associated with strong bootstrap (Fig. 2) and posterior (Fig. 3) support.

The first colonization is by an ancestor (Node 1 in Figs. 2, 3) of the species in *Euconocephalus* giving rise to *Banza nihoa* (Figs. 2, 3), and the second by an ancestor (Node 2 in Figs. 2, 3) of *R. dubia* giving rise to the rest of *Banza* species (Figs. 2, 3). Thus, the genus *Banza* is paraphyletic in the sense that it does not include *R. dubia* and the two *Euconocephalus* species. The genus *Ruspolia* is polyphyletic because the two *Ruspolia* species are from separate lineages with their respective sister taxa. *Banza nihoa* is morphologically, acoustically, behaviorally and ecologically different from the rest of the *Banza* species [11, 15], and was tentatively placed in a different genus [15]. Our phylogeny offers an evolutionary explanation for the differences recorded between *B. nihoa* and the rest of the *Banza* species, *i.e.*, *B. nihoa* is a distinct lineage from the rest of *Banza* species in Hawaii Islands.

The phylogenetic relationship in Fig. 2 is consistent with other studies using 18S and 28S rDNA, cytochrome oxidase II, histone 3, tubulin alpha 1 and wingless genes [16]. Indeed, *Conocephalus* is the sister group of Copiphorinae which splits between *Neoconocephalus* and *Euconocephalus* + *Ruspolia.* The tree is also similar to that in Shapiro et al. [11] if we remove *Ruspolia* and *Euconocephalus* species. However, such removal would give a misleading impression that *Neoconocephalus* is the sister lineage of the *Banza* species in Hawaii Islands and that *Banza* is a monophyletic taxon. This exemplifies the importance of thorough taxon sampling. We should note that *Ruspolia* and *Euconocephalus* are closely related and still have species transferred from one genus to the other. For example, *Euconocephalus indicus* is now considered as a synonym for *Ruspolia indica* [17].

### Dating speciation and phylogeographic events

As the volcanic islands need to emerge from the bottom of the ocean to be colonized (Fig. 1), their emergence dates can serve as maximum ages in molecular dating [18]. For example, if each colonization is directly from the coastal region of Asia, then we may limit the age of Node 1 (Figs. 2, 3) to be no earlier than the age of Nihoa (~ 7.3 mya). Similarly, the age of Node 2 may be limited to no earlier than the age of Kauai (~ 5.8 mya). However, one should be cautious with this approach because Hawaii Islands represent only the youngest ones of the Hawaiian-Emperor seamount chain that sequentially emerged from the Pacific Ocean through volcanic eruptions at a relatively fixed hotspot [3, 19]. The older islands were once large but have shrunk and sunk over time. It is possible that an island older than the current Hawaii Islands was colonized by an ancestral population at Node 1, say 10 mya, before the emergence of any current Hawaiian Islands. Therefore, this ancestral island diverged from the mainland lineage 10 mya. Subsequently, this ancestral lineage colonized younger island as it emerged from the ocean. In this scenario, the age of the younger island should not be used to set the earliest time of Node 1 ancestor because its divergence from the mainland lineage occurred long before the emergence of the younger island. On the other hand, it is reasonable to assume that genetic diversification of katydid lineages among islands could not occur before the emergence of the islands. For example, descending lineages of Node 3 (Figs. 2, 3) evolved and diversified among Kauai and younger islands. We may limit the age of Node 3 as no earlier than the emergence of Kauai (~ 5.8 mya).

#### Dating approach 1

**Fig. 4:**
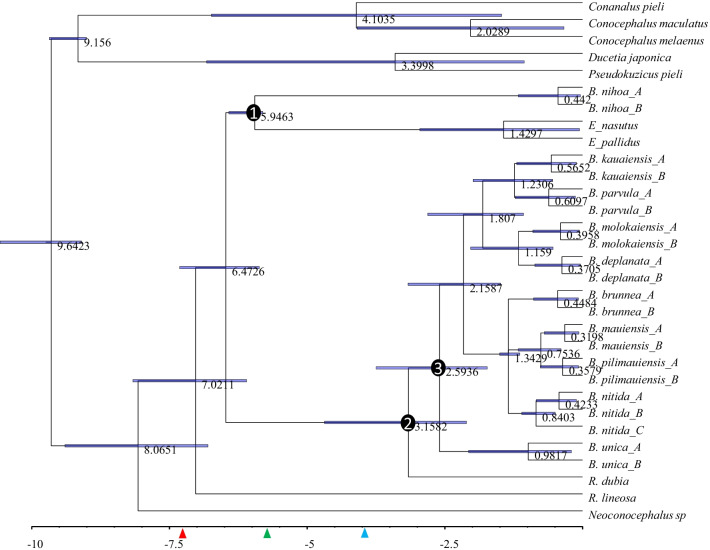
Dated phylogenetic trees from MrBayes. The horizontal scales are in million years. Bars at the internal nodes show the 95% Highest Posterior Densities for date estimates. Numbers near internal nodes show mean dates. The red, green and blue arrowheads indicate the age of Nihoa, Kauai and Oahu, respectively

We dated the ancestors in two ways. The first assumes that all colonization events are independent and directly from the coastal regions of Asia. This limits Node 1 to be no earlier than the emergence of Nihoa (~ 7.3 mya) and Node 2 to be no earlier than the emergence of Kauai (~ 5.8 mya). With these restrictions, the first colonization event that gave rise to *Banza nihoa* was dated to about 6 million years ago (mya) (Fig. 4), after the emergence of Nihoa Island dated at 7.3 [2, 14]. The second colonization event that gave rise to the rest of *Banza* species was dated to 3.2 mya. The oldest lineage from this second colonization event was *B. unica* currently inhabiting Oahu Island that emerged about 3.9 mya [2, 14].

**Fig. 5:**
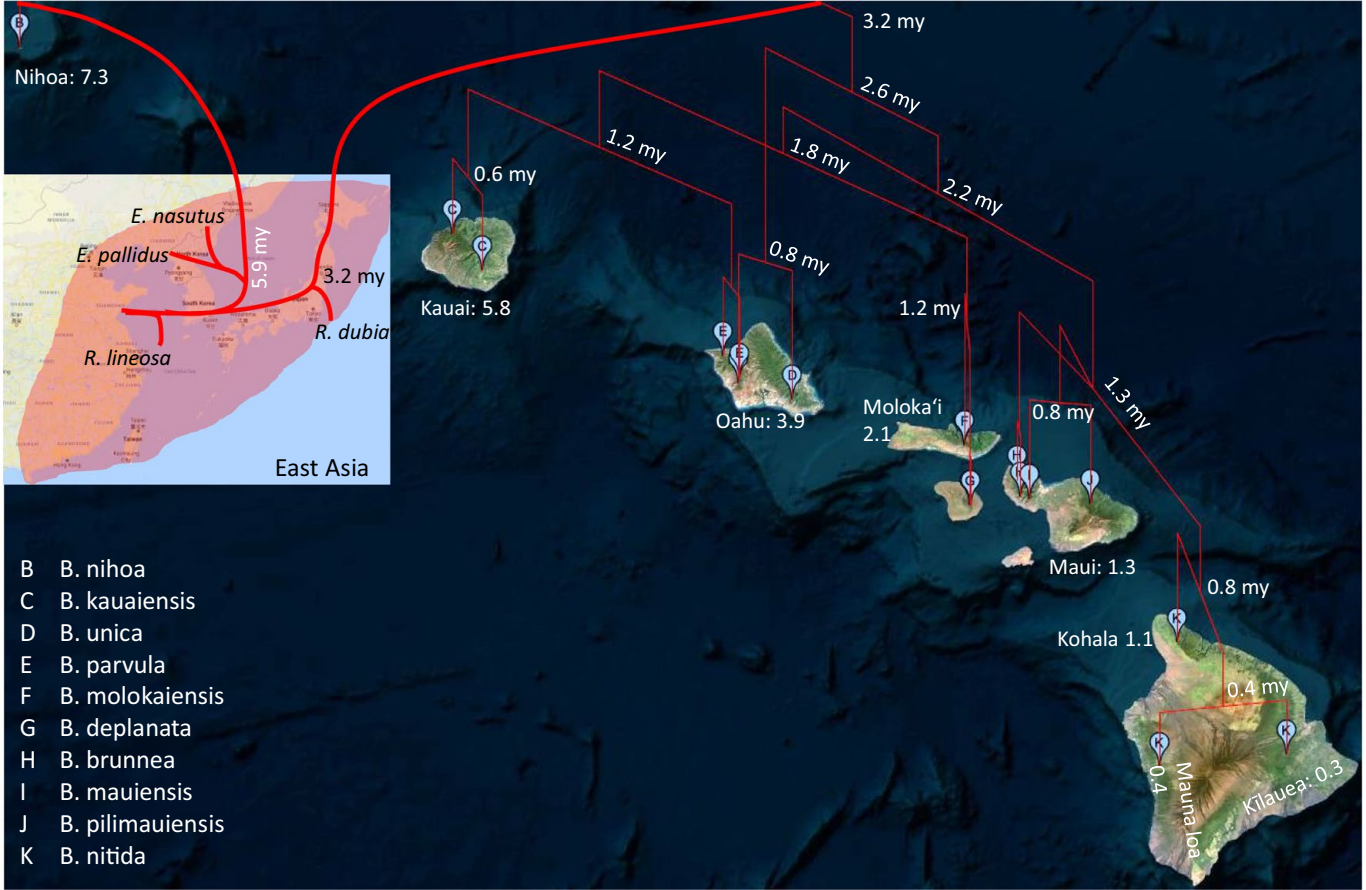
Geophylogeny of Hawaiian katydids and their relatives, generated from PGT [21]. Shaded areas in East Asia inset represents the distribution of *Ruspolia* and *Euconocephalus* on the coastal and island regions. Vertical branches in the geophylogeny of Hawaiian katydids represent branch length. Divergence time in dated internal node is shown in million years (my). The geological age of each island is shown next to the islands. Species names associated with the letters at the tips of the geophylogeny are listed in the lower-left of the figure

Because the specimens of *Banza* species have geographic coordinates, we constructed a geophylogeny of the two colonization events (Fig. 5). From East Asia, a sister lineage of the common ancestor of *Euconocephalus* colonized Nihoa Island, giving rise to *Banza nihoa* about 5.9 million years ago (Fig. 4). As Nihoa Island was dated to have emerged about 7.3 mya, while the first colonization event took place around 5.9 mya, the individuals of this first colonization must have quickly become flightless before Kauai Island, which appeared about 5.8 mya (Fig. 5), became inhabitable. Consequently, *B. nihoa* was confined to Nihoa. Loss or gain of wings occur frequently in the stick insects in which fully winged, partially winged and wingless species originated multiple times independently [20]. We discuss later the benefit and cost of different wing morphs in isolated ocean islands.

The second colonization event was realized by a sister lineage of *R. dubia ca.* 3.2 mya, so the initial colonization could have occurred on Kauai emerged about 5.8 mya. However, only a young *Banza* lineage (*B. kauaiensis*) was found on Kauai. Thus, either the ancient *Banza* lineage from this second colonization have gone extinct on Kauai, or the colonizers first arrived on the next Oahu that emerged 3.9 mya. The most ancient lineage from this second colonization event is *B. unica* found only on Oahu.

This second colonization event was then followed by island-hopping to colonize younger islands. The sister lineage of *B. unica* diverged into two lineages. The northwestern lineage is represented by *B. kauaiensis, B. parvula, B. deplanata,* and *B. molokaiensis* colonizing the older islands (Kauai, Oahu and Molokai)*.* The southeastern lineage consists of *B. nitida, B. mauiensis, B. pilimauiensis,* and *B. brunnea* and colonized the younger islands Maui and Hawaii (Fig. 5). The sequence divergence among the three *B. nitida* specimens are larger than that between *B. kauaiensis* and *B. parvula* or between *B. brunnea* and *B. pilimauiensis* (Figs. 2–3), prompting Shapiro et al. [11] to suggest the existence of cryptic species within *B. nitida*.

#### Dating approach 2

The dating approach above by using the age of Nihoa and Kauai as the maximum age of Node 1 and Node 2 ancestors may not be appropriate. The trees in Figs. 2, 3 indicate that Node 2 could actually be older than Node 1. It is possible that the Node 2 ancestor colonized an older island at time T_2_ and then hopped onto Kauai at T_2_' after its emergence. In this case, T_2_ is earlier than the emergence of Kauai or even earlier than the emergence of Nihoa. For this reason, we should not limit the T_2_ as no earlier than the emergence of Kauai. However, one may reasonably assume that genetic diversification of katydid lineages among islands could not occur before the emergence of the islands. For example, descending lineages of Node 3 (Figs. 2, 3) evolved and diversified among Kauai and younger islands. We may limit the age of Node 3 as no earlier than the emergence of Kauai (~ 5.8 mya).

**Fig. 6:**
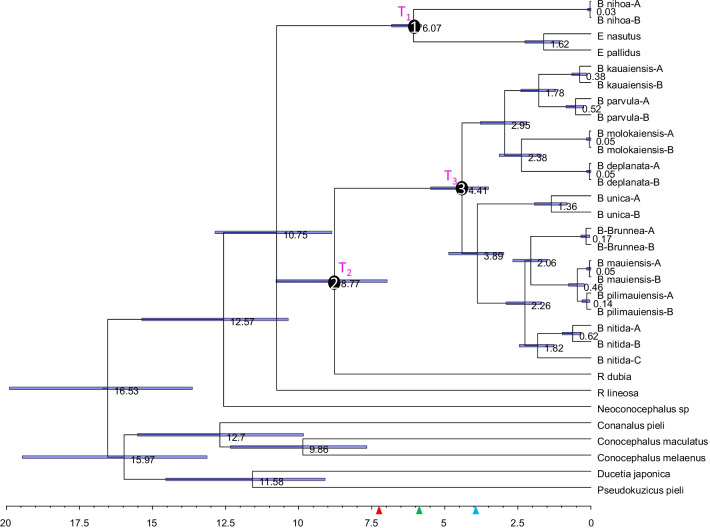
Dated phylogenetic trees from MrBayes without restricting T_2_ with age of Kauai (5.8 mya), but with restriction of T_1_ to be no earlier than 7.3 mya (age of Nihoa) and T_3_ no earlier than 5.8 mya. The horizontal scales are in million years. Bars at the internal nodes show the 95% Highest Posterior Densities for date estimates. Numbers near internal nodes show mean dates. The red, green and blue arrowheads indicate the age of Nihoa, Kauai and Oahu, respectively

This relaxation of age restriction on T_2_ leads to new dating results in Fig. 6. While T_1_ (Fig. 6) is still about 6 mya, T_2_ is now 8.8 mya, earlier than Nihoa. This is consistent with the hypothesis that the Node 2 ancestor diverged from the mainland lineage about 8.8 mya and colonized an older island in the Hawaiian–Emperor seamount chain. This ancient island lineage subsequently colonized Kauai, Oahu and the younger islands as they emerged from the ocean.

One may note a trichotomy in Fig. 6 with three descending lineages (Node 1, Node 2 and *R. lineosa*). This trichotomy is not present in Fig. 4 where Node 2 is constrained by a calibration time. The reason for this trichotomy in Fig. 6 may be visualized from unrooted trees in Figs. 2, 3. Node 4 in Figs. 2, 3 has two descending lineages, Lineage 1 represented by *R. lineosa* and Lineage 2 represented by all other descendants from Node 4. It is easy to see that the distance from Node 4 to *R. lineosa* (D_1_) is smaller than the average distance from Node 4 to the 24 descendants of Lineage 2 (D_2_). Fitting a molecular clock would tend to force D_1_ = D_2_, which favors a shift of Lineage 2 towards the root, and eventually merging the ancestor of Lineage 2 with Node 4 leading to a trichotomy.

**Fig. 7:**
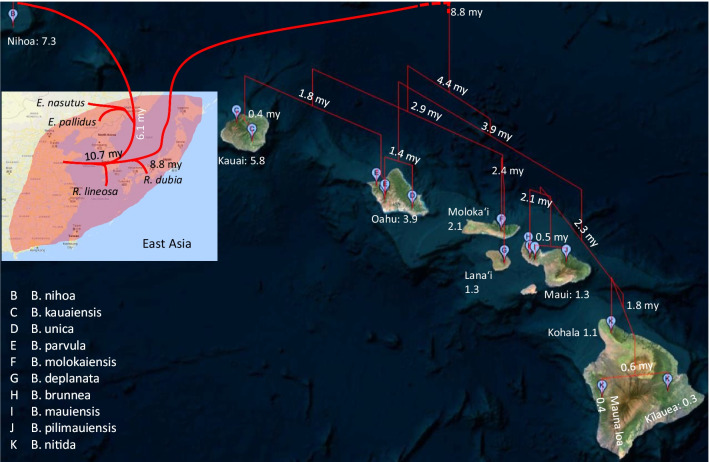
Geophylogeny of Hawaiian katydids and their relatives, generated from PGT [21], with the phylogenetic tree from Fig. 6. The ancestor of *R. dubia* is hypothesized to have landed in an older island along the Hawaiian-Emperor seamount chain and subsequently colonized Kauai when it emerged from the Pacific Ocean. The meaning of numbers and names is the same as in Fig. 5

The dating results in Fig. 6 leads to a different geophylogeny in Fig. 7. By 4.4 mya, this island lineage colonizing Kauai had already diverged into two lineages (referred hereafter as Lineage 1 and Lineage 2). Lineage 1 initially diverged in Kauai and Oahu. Around 2.1 mya when Molokai was formed, Lineage 1 colonized Molokai. We designate the original Lineage 1 on Kauai and Oahu as Lineage 1.1 and those colonized Molokai as Lineage 1.2. Lineage 1.1 speciated into *B. kauaiensis* on Kauai Island and *B. parvula* on Oahu Island. Lineage 1.2 speciated into *B. molokaiensis* on Molokai and *B. deplanata* on Lanai. Note that the divergence time of *Banza* species could be older than the age of the island they inhabit because the katydids arriving at the island could already carry divergent matrilineal mitochondrial DNA.

Lineage 2 initially colonized Oahu and diverged into two lineages, Lineage 2.1 and Lineage 2.2. Lineage 2.1 becomes the extant *B. unica* remaining in Oahu. Lineage 2.2 colonized the newest southeastern islands, i.e., Maui and the Big Island as they emerge from the Pacific Ocean, and speciated into *B. brunnea, B. mauiensis* and *B. pilimauiensis* on the island of Maui, and *B. nitida* o the Big Island. As *B. nitida* has already become flightless, the new island that is forming underwater to the southeastern side of the Big Island most likely would need new colonization by winged mainland populations from the Old World or the New World.

## Discussion

### Long-distance migration, colonization, speciation and extinction

While it remains a matter of debate how the ancestors of Hawaiian katydids crossed the vast span of the Pacific to arrive at the Hawaiian Islands, such long-range migration is possible for two reasons. First, the earth has undergone cold and warm periods. During cold glaciation period, much ocean water was locked on land in the form of thick icesheets, with sea level much lower than it is today. For example, the ice sheet in Antarctica expanded to its maximum around 5.6 mya, contributing to the decrease in sea level and the complete drying-up of the Mediterranean Sea [5]. This corresponds roughly to the inferred colonization time of the Nihoa Island. The thick icesheets on the land and the consequent reduction in sea level imply that many islands under water in the Pacific today were above water level during glaciation period, eliminating the necessity of colonizers flying for thousands of kilometers in a single flight. Second, *Ruspolia* species, *e.g.*, *R. differens,* are strong flyers, comparable to their relatives such as locusts and grasshoppers. For instance, in 1988, swarms of desert locust (*Schistocerca gregaria*) flew across the Atlantic Ocean covering about 5,000 km from West Africa to the Caribbean Islands in ten days, prompting the proposal that the New World *Schistocerca* species were derived from ancestral African lineages [22, 23]. This contrasts with the alternative hypothesis that the common ancestor of *Schistocerca* lived before the separation of Africa and South America and that the Old World *Schistocerca* and New World *Schistocerca* lineages are sister lineages resulting from the separation of the two continents. Only molecular phylogenetics with sufficient taxon sampling and accurate dating can fully resolve such alternative hypotheses. The point we wish to make here, however, is that it is not inconceivable for the Asian ancestors of katydids to cross the ocean and colonize Hawaiian Islands.

The example of transatlantic locust migration by desert locusts above does highlight the uncertainty underlying phylogeographic studies. If such a species becomes established in America and, after millions of years, loses its ability of transatlantic flight, the phylogeographic pattern may mislead researchers to conclude that the species was present before the separation between Africa and America but have evolved very slowly ever since. Similarly, one may hypothesize that extreme storms blowing westward contribute significantly to biogeography in the Pacific islands [10] and gain undeserved support of the hypothesis. For example, if we remove *Euconocephalus* and *Ruspolia* species from Figs. 2, 3, 4, 5, 6, 7, then the pattern would be supportive of this hypothesis because *Banza* species appear to have descended from the New World *Neoconocephalus.* For this reason, we should follow Shapiro et al. [11] by cautioning readers of possible pitfalls in phylogeographic interpretations.

Katydids have colonized Hawaiian Islands many times, but only those with extant lineages are observable. *Conocephaloides hawaiiensis* [24], with fully developed wings and extraordinarily long femurs, may have gone extinct as it had not been found with extensive field sampling [15]. Only a color drawing of *C. hawaiiensis* remains [24], together with a brief description on how it can be distinguished morphologically from *Conocephalus* and *Brachymetopa* species. *Brachymetopa* is a synonym of *Banza*, and *Conocephaloides* species have been assigned to *Euconocephalus*, *Ruspolia* and *Neoconocephalus* [17]. *Euconocephalus remotus* was on IUCN’s Red List of Threatened Species in 1996 and likely has gone extinct [11, 15, 25]. This highlights the urgency of sampling species endemic in Hawaii Islands.

The population decline and extinction of certain Hawaiian katydids may be caused by inadvertently introduced species, such as grasshoppers, that could severely damage vegetation in a large scale [9]. In addition to inadvertently introduced species, many “beneficial” species have been introduced into Hawaiian Islands, starting with the ladybeetle *Novius cardinalis* in 1890 [26, 27]. Unfortunately, thousands of species introduced into Hawaiian Islands were not recorded [27]. A large-scale barcoding project would help identify these introduced species as they should exhibit little genetic differentiation from their respective source populations. It would indeed be a shame for ecologists and evolutionary biologists not to reap scientific understanding from such a large-scale experiment on introduced species.

### Evolution of wing-loss

Given the success of insects, there must be an evolutionary advantage of flight [28]. Such advantage includes the efficiency in finding and locating new food, nesting and mating resources. Limited evidence suggests that such advantages may be greater in males than in females. Whenever there is sexual dimorphism in wings in stick insects, it is always the male that has more wings [20]. However, in spite of the advantage of flight, the loss of flight in insects evolved multiple times in the Hawaiian Islands in 10 of the 11 orders of insects, often with the alate and flightless sister species living side by side [6].

There are two hypotheses invoking a fitness advantage for the loss of flight. Darwin's original proposal [29] is that the chance of being blown into the sea is greater than the benefit of finding better resources. If a population lives in a small, windy and isolated island, then the cost is obviously great and the benefit little. This would predict an inverse relationship between the propensity of wing loss and the size of an island, and he cited the more apterous forms in the small islet Dezerta Grande than the larger Madeira to support his argument. The same association has also been observed in Hawaii Islands. The introduced and fully winged *Schistocerca nitens nitens* took flight more readily in the large Hawaiian Islands than in smaller ones [15]. This relationship between windy and spatially confined environment and insect wing loss was extensively documented subsequently [28, 30–33]. This hypothesis has been extended to include the energetic cost of maintaining wings in a windy environment [34].

The second hypothesis, implicitly alluded to by Roff [28] and Whiting et al. [20], invokes the benefit of occupying vacant niches. Mainland habitats invariably features many non-flying terrestrial and subterranean arthropods. These non-flying arthropods are few in remote islands because of their limited dispersal capability. This implies that the ecological niches typically occupied by such non-flying arthropods are vacant in remote islands. Consequently, there is potential benefit for some insects to lose their wings to fill in such niches. After all, almost all insects, including the strongest fliers, spend much of their life without using wings. This second hypothesis is applicable not only to islands, but also to high-rising mountain peaks with isolated populations. When such an isolated population of non-flying arthropods go extinct, their ecological niches are vacated and not readily filled by other non-flying arthropods living in other isolated peaks. Thus, we expect insects to have an increased propensity to lose their flight and become ground-dwelling in habitats where non-flying arthropods are rare. The two hypotheses outlined above are not mutually exclusive. Rather, they highlight different aspects of potential fitness gain. Hawaiian Islands would serve as an ideal place for evaluating the relative importance of these two hypotheses.

### Can the island population from Hawaii back-colonize the mainland?

One of the reviewers suggested the possibility of *R. dubia* originated as a consequence of back-colonization of Asian mainland. That is, after establishing an island population in Nihoa, some descendants of the original colonizers flew back to the Asian mainland and evolved into *R. dubia.* This suggestion is likely prompted by the observation that species in *Ruspolia* and *Euconocephalus* are often strong fliers. If they could fly from Asian mainland to Hawaii, then it is theoretically possible to make a return trip, especially because extreme storms tend to blow westward [10].

Three lines of reasoning would go against such a back-colonization hypothesis. First, even fully winged insects in Hawaii Islands appear to refrain from taking flight, with the consequence that few insect species are found in more than one island [15]. This is consistent with the hypothesized cost of alate insect forms being blown by wind into open water and drowned. The recently introduced grasshopper (*Schistocerca nitens nitens*) is fully winged and can still fly in larger islands but rarely do so in small islands such as in Nihoa [15]. Second, long-distance migration in insects is typically proceeded by a very large population with the consequent swarming behavior. Insect populations on remote islands of limited sizes typically do not reach a population size large enough for swarming, so back colonization is unlikely. Third, in contrast to Hawaiian Islands which emerged as volcanic islands with hardly any biodiversity, the mainland habitat is typically rich in a variety of insect species. Therefore, even if a few exhausted katydids from Hawaii Islands made their way to the mainland, they would be unlikely to win against established and well-adapted mainland competitors.

### The importance of having large-scale phylogeographic data

Phylogeographic studies require dense sampling because the fundamental question addressed by phylogeography is why a species X is present in Area A but not in Area B. The distribution pattern of species X could have four causes. First, X has limited dispersal capability and has never had a chance to arrive in Area B. Second, X did disperse to Area B but could not survive and reproduce in Area B. Third, X has arrived in Area B, survived and reproduced, but has evolved into species Y. Fourth, X has arrived in Area B, survived and reproduced, but was not encountered and sampled by the researcher.

This conceptual framework is relevant in interpreting the distribution pattern of *Banza* species after the second colonization event (Figs. 2, 3, 4, 5, 6, 7). These colonizers must have first landed in Kauai and then Oahu because the younger islands have not emerged yet. One therefore would expect the highest genetic diversity and the most ancient *Banza* lineages to be found in either Kauai or Oahu. However, the two *B. kauaiensis* specimens found in Kauai are neither ancient nor genetically diverse. The extensive sample collection [15] decreased the plausibility of the hypothesis that ancient *Banza* lineages are still present in Kauai but not sampled. One may therefore conclude that the ancient *Banza* lineages on Kauai had gone extinct and the island was recolonized by recently derived *B. kauaiensis*. One cannot reach such a conclusion without the extensive sample collection [15].

## Conclusions

We have shown here how phylogeographic analysis could help resolve alternative hypotheses on the origin and evolution of species colonizing a new environment and how it is crucial to have sufficient taxon sampling. Our results revealed the origin of Hawaiian *Banza* katydids by two colonization events, both by katydids from the Old World rather than from the New World. The first colonization was by an ancestral lineage of *Euconocephalus* about 6 mya after the formation of Nihoa about 7.3 mya, giving rise to *B. nihoa.* The second colonization was by a relative of *Ruspolia dubia* giving rise to the rest of *Banza* species in two major lineages, one lineage inhabiting and diverging on the older northwestern islands and the other on the newer southwestern islands. The phylogenetic pattern revealed by our analysis suggests the need of an extensive revision of the taxonomic status of species in *Euconocephalus, Ruspolia* and *Banza.*

## Methods

### Sequence retrieval

The mitochondrial *COX1* and *CYTB* genes for the 23 specimens including ten species of Hawaiian katydids (genus *Banza*) and three outgroup species were downloaded from NCBI (see Additional file 2: Table S1 for accession numbers). Sequences from three sets of potential direct ancestors belonging to the family Tettigoniidae, subfamily Conocephalinae and tribe Copiphorini were also retrieved: (1) *Ruspolia dubia* and *R. lineosa*; (2) six *Euconocephalus indica* individuals; and (3) one belonging to *Neoconocephalus*. The first two species are distributed in eastern and southeastern Asia, including the coastal regions, while *Neoconocephalus* species are distributed in America, including western coastal regions [35].

As outgroups, we use three katydid species belonging to the same subfamily but a different tribe (Conocephalini): *Conanalus pieli, Conocephalus maculatus,* and *Conocephalus melaenus.* Also included are two phylogenetically more distant katydid species, *Ducetia japonica* of the subfamily Phaneropterinae, and *Pseudokuzicus pieli* of the subfamily Meconematinae.

### Sequence alignment, dating, and phylogeographic analyses

Complete mitochondrial genomes (mtDNAs) were parsed with DAMBE [36, 37]. The *COX1* and *CYTB* sequences were aligned using MAFFT [38] with the most accurate LINSI option (‘–localpair’ and ‘–maxiterate = 1000’). Consistent with the mostly non-recombining nature of the mtDNA, the two mitochondrial genes have a concordant phylogenetic signal [11], so that initial phylogenetic analyses were done on the concatenated sequences.

For phylogenetic reconstruction with PhyML v3.3 [39] and RAxML [40], we used jModelTest v.2.1.10 to select the best substitution model [41] based on an estimated BIONJ tree [42] under each model. Table S2 shows the log-likelihood for different models based on the BIONJ tree, with four discrete rate categories for approximating a continuous gamma distribution [43]. For PhyML, the tree improvement option (‘-s’) was set to ‘BEST’ (best of NNI and SPR search). The ‘-o’ option was set to ‘tlr’ which optimizes the topology, the branch lengths and rate parameters. RAxML performs 1000 rapid bootstrap inferences and a thorough ML search. Geophylogenies (i.e., phylogenetic trees mapped onto a geographic map to highlight the spatial and temporal partition of biodiversity) were generated from PGT software [21].

For Bayesian phylogenetic reconstruction with MrBayes v3.2.7 [44], the GTR substitution model was used with gamma-distributed rate variation across sites, and with or without estimating a proportion of invariant sites. For all Bayesian analyses, the run length was increased until the standard deviation of split frequencies is less than 0.01. The sump command shows PSRF (potential scale reduction factor) varying from 1.000 to 1.004, and average ESS (effective sample size) varying from 530 to 1054, for rate and frequency parameters. The resulting cladogram with the posterior probabilities and the phylogram with mean branch lengths were visualized in FigTree [45].

In order to date these phylogeographic patterns, relaxed molecular clock analyses were performed. We used MrBayes v3.2.7 [44] with the independent gamma rates (IGR) model. The clock was calibrated based off the timing of the volcanic emergence of each island, and hence did not employ minimum ages as is usually the case [18], but *maximum* ages by means of unform distributions. Two dating approaches were used, differing in calibration priors. The first restricts the age of Node 1 (Figs. 2, 3) to no earlier than the emergence of Nihoa (~ 7.3 mya) and the age of Node 2 (Figs. 2, 3) no earlier than the emergence of Kauai (~ 5.8 mya). The second removed the restriction on Node 2 because the trees in Figs. 2, 3 suggest that the second colonization may involve the colonization of an island older than the current Hawaii Islands, with the ancestral island lineage colonizing Kauai later. Because the descendants of Node 3 (Figs. 2, 3) have been observed only in Kauai and younger islands, it is assumed that their genetic diversification among the island species cannot be earlier than the emergence of Kauai (~ 5.8 mya). Figures 4, 5 are from the first dating approach and Figs. 6, 7 from the second dating approach. For all dating analysis, the GTR + Γ substitution model was used, without estimating the proportion of invariant sites.

## Supplementary Information

**Additional file 1.** Aligned sequences, with COX1 and CytB separately aligned and then concatenated.

**Additional file 2: Table S1. ** Accession numbers of the sequences used in this study. Species isolates / vouchers, where applicable, are shown between parentheses. Accession numbers are shown for the *COX1* and *cytb* genes. Two accession numbers are for partial *COX1* and *CYTB* sequences, respectively; complete mitochondrial sequences with full-length *COX1* and *CYTB* sequences were indicated by a single accession number. **Table S2.** Model selection results. Twelve models were tested, with Ã components based off the four-category discretization. Number of parameters (*p*), Akaike Information Criterion (AIC), and distance from best (minimal) AIC score (ÄAIC) are shown.

## Data Availability

All data needed to duplicated results in the paper were retrieved from NCBI (https://ncbi.nlm.nih.gov/) and BOLD (https://www.boldsystems.org/). Both are public databases open to everyone. All accession numbers have been provided in Additional file 2: Table S1. Aligned sequences are included as Additional file 1.

## Abbreviations

GTR + Γ: General time reversible substitution model with gamma-distributed rates over sites
GTR + Γ + I: General time reversible substitution model with gamma-distributed rates over sites and a proportion of invariant sites
PGT, PhyML, RAxML, MAFFT, MrBayes: Names of software packages
