# Supplementary material for: Testing alternative hypotheses on the origin and speciation of Hawaiian katydids
Source: BMC Ecol Evol. 2022 Jun 22;22:83. doi: 10.1186/s12862-022-02037-2 (PMC9215005; doi:10.1186/s12862-022-02037-2)
Supplement: Supplementary file 2 — Additional file 2: Table S1. Accession numbers of the sequences used in this study. Species isolates / vouchers, where applicable, are shown between parentheses. Accession numbers are shown for the COX1 and cytb genes. Two accession numbers are for partial COX1 and CYTB sequences, respectively; complete mitochondrial sequences with full-length COX1 and CYTB sequences were indicated by a single accession number. Table S2. Model selection results. Twelve models were tested, with Ã components based off the four-category discretization. Number of parameters (p), Akaike Information Criterion (AIC), and distance from best (minimal) AIC score (ÄAIC) are shown. [file 12862_2022_2037_MOESM2_ESM.docx]

Table S1. Accession numbers of the sequences used in this study. Species isolates / vouchers, where applicable, are shown between parentheses. Accession numbers are shown for the *COX1* and *cytb* genes. Two accession numbers are for partial *COX1* and *CYTB* sequences, respectively; complete mitochondrial sequences with full-length *COX1* and *CYTB* sequences were indicated by a single accession number.

| Species | ACCN(1) | Distribution |
| --- | --- | --- |
| *Banza nihoa* (A) | DQ649491, DQ649515 | Nihoa |
| *B. nihoa* (B) | DQ649492, DQ649516 | Nihoa |
| *B. kauaiensis* (A) | DQ649483, DQ649507 | Kauai |
| *B. kauaiensis* (B) | DQ649484, DQ649508 | Kauai |
| *B. unica* (A) | DQ649501, DQ649525 | Oahu |
| *B. unica* (B) | DQ649502, DQ649526 | Oahu |
| *B. parvula* (A) | DQ649497, DQ649521 | Oahu |
| *B. parvula* (B) | DQ649498, DQ649522 | Oahu |
| *B. molokaiensis* (A) | DQ649487, DQ649511 | Molokai |
| *B. molokaiensis* (B) | DQ649488, DQ649512 | Molokai |
| *B. deplanata* (A) | DQ649481, DQ649505 | Lanai |
| *B. deplanata* (B) | DQ649482, DQ649506 | Lanai |
| *B. brunnea* (A) | DQ649479, DQ649503 | West Maui |
| *B. brunnea* (B) | DQ649480, DQ649504 | West Maui |
| *B. mauiensis* (A) | DQ649485, DQ649509 | West Maui |
| *B. mauiensis* (B) | DQ649486, DQ649510 | West Maui |
| *B. pilimauiensis* (A) | DQ649499, DQ649523 | East Maui |
| *B. pilimauiensis* (B) | DQ649500, DQ649524 | East Maui |
| *B. nitida* (A) | DQ649493, DQ649517 | Hawaii |
| *B. nitida* (B) | DQ649495, DQ649519 | Hawaii |
| *B. nitida* (C) | DQ649494, DQ649518 | Hawaii |
| *R. lineosa* | NC_033991 | East Asia |
| *R. dubia* | NC_009876 | East Asia |
| *Neoconocephalus sp* | DQ649489, DQ649513 | America |
| *Euconocephalus nasutus* | NC_053383 | Asia |
| *E. pallidus* | MW009066 | Asia |
| *Conanalus pieli* | KX057724 | East Asia |
| *Conocephalus maculatus* | KM244677 | Asia/Africa |
| *Conocephalus melaenus* | NC_033988 | Asia |
| *Ducetia japonica* | KU885974 | Asia/Australia |
| *Pseudokuzicus pieli* | KX057712 | East Asia |

Table S2. Model selection results. Twelve models were tested, with  components based off the four-category discretization. Number of parameters (*p*), Akaike Information Criterion (AIC), and distance from best (minimal) AIC score (AIC) are shown.

| Model | –log-likelihood | *p* | AIC | AIC |
| --- | --- | --- | --- | --- |
| GTR+ | 16,990.2431 | 76 | 34,132.4862 | 0.0000 |
| HKY+ | 17,060.1138 | 72 | 34,264.2276 | 131.7413 |
| SYM+ | 17,347.3534 | 73 | 34,840.7068 | 708.2205 |
| F81+ | 17,823.4407 | 71 | 35,788.8813 | 1,656.3951 |
| K80+ | 17,905.3226 | 69 | 35,948.6452 | 1,816.1589 |
| JC+ | 18,496.7471 | 68 | 37,129.4942 | 2,997.0079 |
| GTR | 18,728.7194 | 75 | 37,607.4387 | 3,474.9525 |
| SYM | 18,893.7831 | 72 | 37,931.5662 | 3,799.0800 |
| HKY | 19,098.0537 | 71 | 38,338.1074 | 4,205.6212 |
| K80 | 19,618.1694 | 68 | 39,372.3387 | 5,239.8525 |
| F81 | 19,864.8654 | 70 | 39,869.7308 | 5,737.2445 |
| JC | 20,343.2848 | 67 | 40,820.5695 | 6,688.0833 |
